# Phenotypic screening of signaling motifs that efficiently induce cell proliferation

**DOI:** 10.1038/s41598-023-42378-6

**Published:** 2023-09-20

**Authors:** Kirato Umene, Teruyuki Nagamune, Masahiro Kawahara

**Affiliations:** 1 Department of Chemistry and Biotechnology, Graduate School of Engineering, The University of Tokyo, 7-3-1 Hongo, Bunkyo-ku, Tokyo, 113-8656 Japan; 2 Laboratory of Cell Vaccine, Microbial Research Center for Health and Medicine (MRCHM), National Institutes of Biomedical Innovation, Health and Nutrition (NIBIOHN), 7-6-8 Saito-Asagi, Ibaraki-shi, Osaka 567-0085 Japan

**Keywords:** Biotechnology, Molecular engineering, Protein design, Synthetic biology, Genetic engineering

## Abstract

Since cell proliferation is one of the fundamental cell fates, artificial control of cell proliferation based on a receptor-engineering approach is increasingly important in therapeutic and industrial applications. Since the signal transduction properties of cytokine receptors are greatly influenced by the amino acid sequence of tyrosine motifs, here we develop a phenotypic screening approach that can directly select cell proliferation-inducing tyrosine motifs from a synthetic library. In the tyrosine motif library, amino acid sequences around the tyrosine are randomized to attain diverse binding patterns of signaling molecules. Theoretically, engineered receptors with distinct tyrosine motifs would activate signaling molecules in diverse patterns. Thus, we investigated whether tyrosine motif sequences capable of inducing cell proliferation could be selected from the cellular library expressing the motif-engineered receptors. Consequently, the selected motifs induced similar levels of cell proliferation compared to the cytoplasmic signaling domain of a native receptor. The motif-screening system was applicable to cells that may differentiate or proliferate depending on cytokine signals. To our best knowledge, this is the first report demonstrating phenotypic screening of tyrosine motifs in living cells. Our approach would open up new possibilities in the field of artificial control of cell fate based on signal transduction engineering.

## Introduction

Cells undergo various cell fates in response to external stimuli, which regulate homeostasis of the body. Since cell proliferation is one of the fundamental cell fates, artificial control of cell proliferation is extremely important in therapeutic and industrial applications that utilize human and mammalian cells^1^. In regenerative medicine and cell therapy, cells often need to be expanded for securing a sufficient number of therapeutic cells for the treatment^2^. In protein production, since both the number of cells and protein productivity per cell profoundly affect the product yield, breeding fast-proliferating high-producer cell lines is a typical way of process optimization for industrial applications.

Cell proliferation is controlled via signal transduction pathways, which are initiated by activating cytokine receptors expressed on the plasma membrane with their specific ligands^3^. There are mainly two approaches for artificially controlling signal transduction. One approach is to screen or rationally design surrogate ligands for cytokine receptors, which could alter or fine-tune the signaling properties by inducing receptor conformations different from those with natural ligands^4–8^. This approach, however, has a limitation that the signaling properties intrinsically depend on endogenous receptors. Reciprocally, the other approach is to engineer cytokine receptors on a functional domain or motif basis, which could arbitrarily design or create the signaling properties in a bottom-up fashion^9–12^. Chimeric antigen receptors can redirect and activate T cells toward specific cancers because the receptors are composed of a single-chain Fv antibody against a target cancer antigen in the extracellular domain and a co-stimulatory/CD3ζ chimeric signaling region in the cytoplasmic domain^13^. Furthermore, fusing a specific signaling molecule-binding motif to a chimeric antigen receptor enhances anticancer activity in mice^14^. Thus, the receptor-engineering approach is a powerful tool to customize cell fate for therapeutic applications.

In order to engineer receptors with desired functions, we need insights into the signal transduction mechanism of natural cytokine receptors. The cytoplasmic domain of type I cytokine receptors function as a scaffold for signal transduction, and consist of box1/box2 regions and tyrosine motifs for recruitment of a tyrosine kinase JAK and signaling molecules, respectively. Upon ligand binding, cytokine receptors are oligomerized, leading to activation of pre-associated JAK and subsequent phosphorylation of tyrosine residues in the cytoplasmic domain of the receptors. Then, signaling molecules are recruited to the phosphotyrosine and its surrounding amino acid residues (denoted as a tyrosine motif) via the Src homology 2 (SH2) or phosphotyrosine binding (PTB) domain. Consequently, the signaling molecules are phosphorylated by JAK, leading to activation of downstream signaling cascades^3^. Thus, the signal transduction properties of cytokine receptors are greatly influenced by the amino acid sequence of tyrosine motifs, to which each signaling molecule has a preference for binding at micromolar to sub-micromolar order of dissociation constant^15^. Since human and mouse commonly have 120 types of SH2 domains embedded in 110 types of signaling molecules, tyrosine motifs would interact with a pool of signaling molecules at different patterns of affinities^16^. While investigators have endeavored to characterize the binding properties of SH2 / PTB domains to tyrosine motifs, many unknowns hamper the rational design of tyrosine motifs with a desired activation pattern of signaling molecules.

On another aspect, there still lacks the overall landscape about how various signaling molecules contribute to cell fate such as proliferation and differentiation in diverse cell types. In other words, the combination and activation level of signaling molecules for inducing desired cell fate remain to be clearly elucidated. This aspect also limits the rational design of engineered receptors for desirably controlling cell fate.

**Figure 1:**
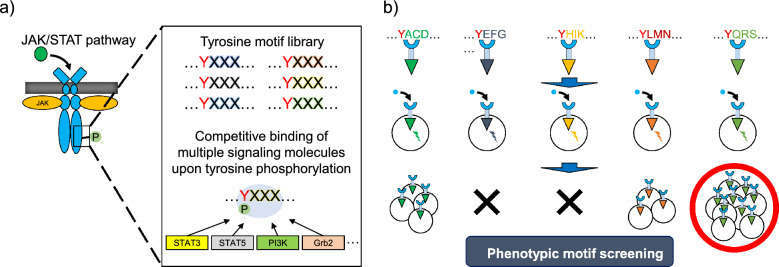
(**a**) Outline of a tyrosine motif library. The library can be generated by introducing random-mutation to residues downstream of tyrosine. Each motif sequence could competitively recruit multiple signaling molecules upon tyrosine phosphorylation and induce different activation patterns of signaling molecules, leading to different cell fates consequently. (**b**) Outline of screening motifs that induce cell proliferation. A particular activation pattern of signaling molecules is attained upon addition of a specific ligand to each cell expressing a clonal chimeric receptor incorporating a randomized motif sequence. Proliferation-inducible motif sequences can be identified by picking up proliferative clones.

To propose a solution for these issues, here we focus on cell proliferation among various cell fates and develop a phenotypic screening approach that can directly select cell proliferation-inducing tyrosine motifs from a synthetic library. In the tyrosine motif library, amino acid sequences around the tyrosine are randomized to attain diverse binding patterns of signaling molecules (Fig. 1a). An engineered receptor library that clonally includes each sequence of the tyrosine motif library is generated and expressed in target cells to create a cellular library (Fig. 1b). Theoretically, engineered receptors with distinct tyrosine motifs would activate signaling molecules in diverse patterns. Thus, we investigate whether tyrosine motif sequences capable of inducing cell proliferation could be selected from the cellular library expressing the motif-engineered receptors.

In previous studies, exhaustive in vitro binding experiments using phosphotyrosine-containing peptide arrays clearly demonstrated that the SH2 domains derived from multiple signaling molecules were able to bind to each phosphotyrosine motif^15^. Therefore, signaling molecules with the SH2 domains usually compete for each phosphotyrosine motif, which results in activation of multiple downstream effectors. In this study as well, we aim to screen for novel motif sequences that simultaneously activate multiple signaling molecules that are important for inducing cell proliferation.

In recent years, high-throughput screening of T cell receptor has intensively been studied and is revolutionizing the field of receptor screening^17^. A recent study has developed a high-throughput screening method that can simultaneously determine the antigen specificities and gene sequences of T cell receptors using antigens fused with DNA barcodes^18^. In addition, phenotypic screening methods based on cell surface markers and reporter assays using high-throughput instruments have also been reported^19,20^. These methods could also be applied to rapid screening of synthetic receptors that induce desired cellular phenotype. However, there are many cell proliferation markers, each of which differently contributes to cell proliferation depending on cell types. In addition, several days are usually required for sufficiently inducing cell proliferation. Therefore, selecting cells directly based on proliferation as presented in this study would be simpler and more reliable in order to screen for synthetic receptors that efficiently induce cell proliferation.

## Results

### Engineering a receptor scaffold for screening a tyrosine motif library

**Figure 2:**
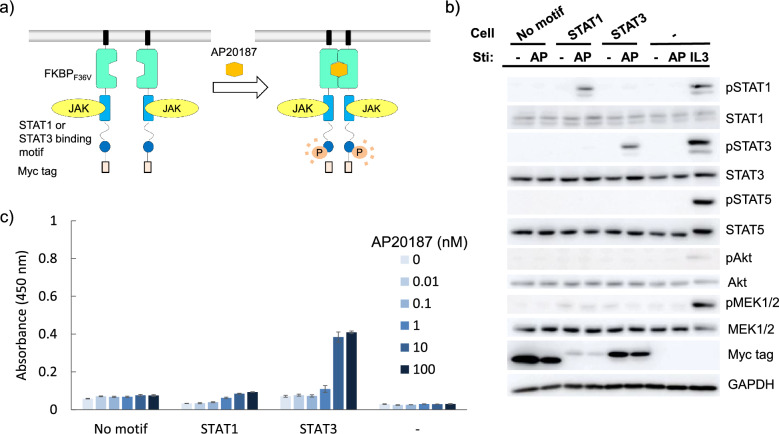
(**a**) Activation mechanisms of a rationally designed receptor scaffold. Monomeric inactive receptors are dimerized by addition of AP20187, which is a specific dimerizer of FKBP_F36V_. This induces activation of a tyrosine kinase JAK, phosphorylation of the tyrosine residue within a tyrosine motif, and activation of signaling molecules corresponding to the properties of the tyrosine motif. (**b**) Western blotting to detect phosphorylation of intracellular signaling molecules. Ba/F3 transductants expressing the receptor constructs fused with no motif, STAT1- and STAT3-binding motifs were depleted in the absence of IL-3 and stimulated for 15 min with/without 10 nM AP20187 or with 1 ng/mL IL-3. Cell lysate was subjected to SDS-PAGE and western blotting. Blots with Myc tag and GAPDH represent receptor expression levels and loading controls, respectively. Uncropped blot images are provided in Supplementary Information (Supplementary Fig. 3). (**c**) Growth assay. Cells were cultured with the indicated concentrations of AP20187 for 3 days with the initial cell density of 1.0 × 10^5^ cells/mL. Cell growth levels were measured by a colorimetric assay. The data are represented as mean ± SE (n = 3, biological replicates).

In order to screen a tyrosine motif library, we rationally engineered a receptor scaffold which could phosphorylate an embedded tyrosine motif and subsequently the on-target signaling molecules in response to an orthogonal ligand (Fig. 2a). The receptor was composed of a myristoylation signal sequence for localization at the plasma membrane, FKBP_F36V_ that homodimerizes with a synthetic ligand AP20187, the JAK-binding domain of thrombopoietin receptor (c-mpl), a (Gly_4_Ser)_3_ linker, a tyrosine motif, and a myc tag. The receptor would therefore be activated by addition of AP20187 through a mechanism that mimics type I cytokine receptors. To prove the concept, a STAT1 or STAT3-binding motif was fused to the receptor as the tyrosine motif. We employed an IL-3-dependent Ba/F3 cell line, which proliferates ligand-dependently also when a variety of type I cytokine receptor variants are exogenously expressed^21–24^. This fact may indicate that Ba/F3 cells could adopt many activation patterns of signaling molecules for their proliferation. Ba/F3 cells were retrovirally transduced with each of the receptors, followed by drug resistance selection to establish stable transductants. The cells were then stimulated by AP20187, followed by western blot analysis to examine whether the on-target signaling molecule could be phosphorylated according to the tyrosine motif embedded in the receptors. As expected, the receptor with the STAT1- or STAT3-binding motif phosphorylated the corresponding STAT protein in response to AP20187, while the receptor without any motifs phosphorylated neither of the STATs (Fig. 2b). We also investigated whether the receptors could trigger AP20187-dependent cell proliferation. The assay revealed that the receptor with the STAT3-binding motif clearly induced AP20187-dependent proliferation, while the receptor with the STAT1-binding motif did not (Fig. 2c). The result is consistent with our recent study showing that STAT3 or STAT5, but not STAT1, promotes proliferation of Ba/F3 cells^25,26^. These results indicate that the engineered receptor scaffold activates signaling molecules bound to the tyrosine motif in response to the synthetic ligand AP20187.

### Screening a tyrosine motif library in Ba/F3 cells

**Figure 3:**
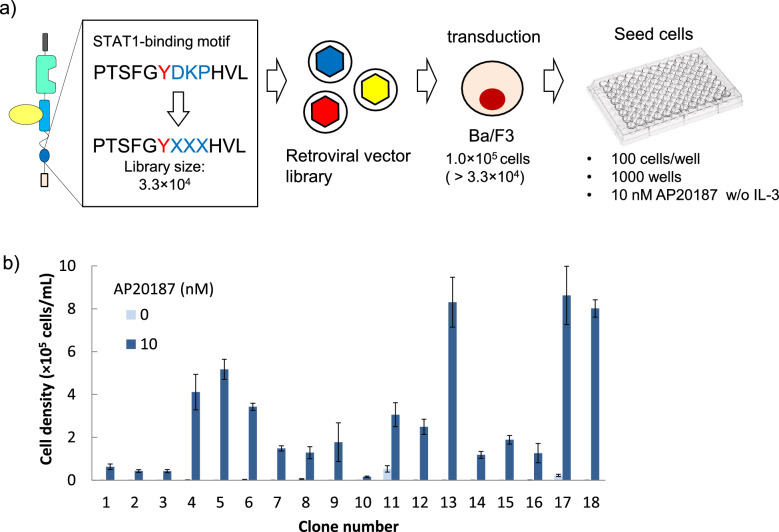
(**a**) Experimental outline of tyrosine motif screening. Three amino acid residues downstream of the tyrosine residue of the STAT1-binding motif were randomized by PCR using a degenerate primer. Ba/F3 cells were transduced with the retroviral vector library, seeded on 96-well plates at 100 cells/well × 1000 wells, and cultured with 10 nM AP20187. (**b**) Growth assay of the screened cellular clones. Cells were cultured with 0 or 10 nM AP20187 for 4 days with the initial cell density of 4.0 × 10^4^ cells/mL. Viable cell densities were measured by flow cytometry. The data are represented as mean ± SE (n = 3, biological replicates).

Since the receptor with the STAT1-binding motif hardly induced cell proliferation (Fig. 2c), the receptor would be appropriate as a template clone for constructing a library, which will be selected on cell proliferation. It has been reported that three amino acid residues downstream of the tyrosine within tyrosine motifs (Y + 1, + 2, and + 3) mainly determine the binding specificity of signaling molecules^15^. With these knowledges in mind, we constructed a tyrosine motif library by randomizing the three residues with PCR using the retroviral plasmid encoding the receptor with the STAT1-binding motif as a template (Fig. 3a). Ba/F3 cells were transduced with the retroviral library, followed by flow cytometric analysis to estimate the transduction efficiency by enhanced green fluorescent protein (EGFP), which was bicistronically encoded in the retroviral library. The estimated transduction efficiency was 1.1%, which was low enough to secure monoclonal transduction of the library into the cells. The resultant cellular library was maintained in the presence of the original proliferation factor IL-3.

For screening, the cellular library was washed to remove IL-3 and cultured in a selection medium containing 10 nM AP20187 for activating the motif-randomized receptors. To easily identify fast-proliferating cellular clones, the cellular library was seeded into multiple 96-well plates. As a result, 18 cellular clones were successfully obtained until 12 days of culture. The cellular clones were then subjected to a cell proliferation assay to examine the response to the ligand AP20187. The result showed that the cellular clones proliferated in an AP20187-dependent manner although the levels were different among the clones (Fig. 3b).

**Figure 4:**
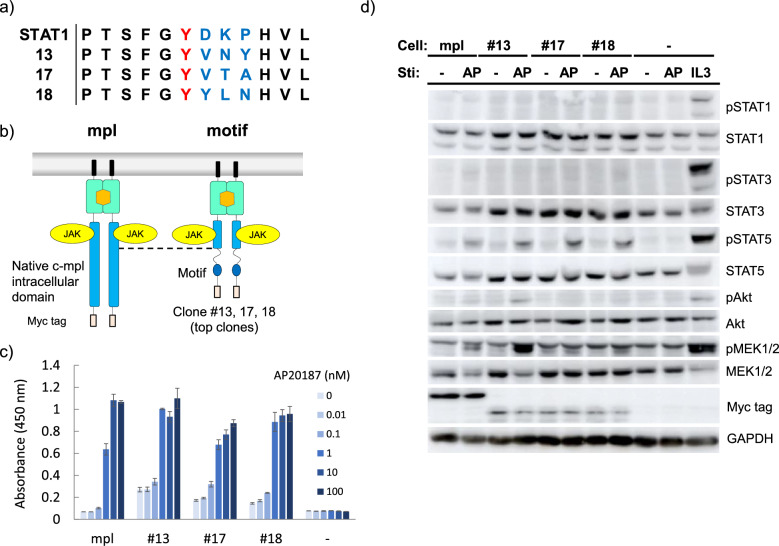
(**a**) The motif sequence of the top-3 proliferative clones. (**b**) Illustration of the control receptor which harbors the intracellular domain of native c-mpl. (**c**) Growth assay. Cells were cultured with the indicated concentrations of AP20187 for 3 days with the initial cell density of 4.0 × 10^4^ cells/mL. Cell growth levels were measured by a colorimetric assay. The data are represented as mean ± SE (n = 3, biological replicates). (**d**) Western blotting to detect phosphorylation of intracellular signaling molecules. Ba/F3 transductants expressing the indicated receptor constructs were depleted in the absence of IL-3 and stimulated for 15 min with/without 10 nM AP20187 or with 1 ng/mL IL-3. Cell lysate was subjected to SDS-PAGE and western blotting. Blots with Myc tag and GAPDH represent receptor expression levels and loading controls, respectively. Uncropped blot images are provided in Supplementary Information (Supplementary Fig. 4).

To characterize the obtained clones in more detail, three fast-proliferating clones (#13, #17, and #18) were chosen. Genomic PCR and subsequent sequencing revealed that the three clones had different sequences at the randomized tyrosine motif site (Fig. 4a). To ensure that these tyrosine motif sequences induce cell proliferation, we subcloned the three motif sequences and additionally the cytoplasmic domain of native receptor c-mpl into the receptor expression plasmid, with each of which Ba/F3 cells were retrovirally transduced to establish drug-resistant stable cell lines (Fig. 4b). Intriguingly, the cell proliferation assay showed that all of the three clones exhibited comparable levels of cell proliferation to the native receptor with similar sensitivity to the ligand AP20187 (Fig. 4c). Signaling analysis showed that activation of STAT5 was commonly observed for all of the receptors (Fig. 4d). While #13 activated higher levels of MEK and Akt compared to the others, the proliferation rate was similar to those of the others. These results proved that tyrosine motif sequences capable of inducing proliferation of Ba/F3 cells were successfully selected from the tyrosine motif library.

### Screening a tyrosine motif library in 32Dcl3 cells

**Figure 5:**
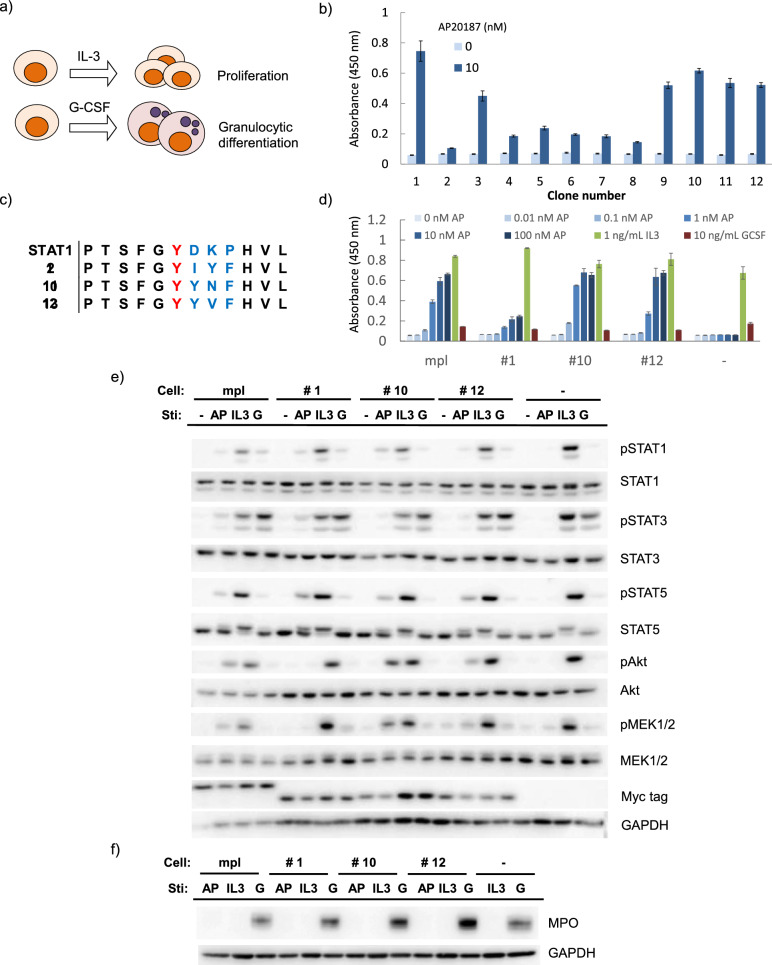
(**a**) Illustration of the properties of 32Dcl3 cells, in which proliferation is induced with IL-3, whereas differentiation is induced with G-CSF. (**b**) Growth assay of the screened cellular clones. Cells were cultured with 0 or 10 nM AP20187 for 5 days with the initial cell density of 1.0 × 10^5^ cells/mL. Cell growth levels were measured by a colorimetric assay. The data are represented as mean ± SE (n = 3, biological replicates). (**c**) The motif sequence of the top-3 proliferative clones. (**d**) Growth assay. Cells were cultured with the indicated concentrations of AP20187, 1 ng/mL IL-3, or 10 ng/mL G-CSF for 3 days with the initial cell density of 2.0 × 10^5^ cells/mL. Cell growth levels were measured by a colorimetric assay. The data are represented as mean ± SE (n = 3, biological replicates). (**e**) Western blotting to detect phosphorylation of intracellular signaling molecules. 32Dcl3 transductants expressing the indicated receptor constructs were depleted in the absence of IL-3 and stimulated for 15 min with/without 10 nM AP20187, with 1 ng/mL IL-3, or with 10 ng/mL G-CSF. Cell lysate was subjected to SDS-PAGE and western blotting. Blots with Myc tag and GAPDH represent receptor expression levels and loading controls, respectively. Uncropped blot images are provided in Supplementary Information (Supplementary Fig. 5). (**f**) Western blotting to detect expression levels of MPO. 32Dcl3 transductants expressing the indicated receptor constructs were cultured with 10 nM AP20187, 1 ng/mL IL-3, or 10 ng/mL G-CSF for 5 days with the initial cell density of 2.0 × 10^5^ cells/mL. For parental cells (-), the lane with 10 nM AP20187 is lacking because the cells died out in the condition. Cell lysate was subjected to SDS-PAGE and western blotting. Blots with GAPDH represent loading controls. Uncropped blot images are provided in Supplementary Information (Supplementary Fig. 6).

To further verify the applicability of this screening system, we employed a myeloid progenitor cell line 32Dcl3, which not only proliferate in response to IL-3 but also differentiate into granulocytes when stimulated with G-CSF^27,28^. Unlike Ba/F3 cells, 32Dcl3 cells may have a narrower window for cell proliferation because different cytokine signals induce different cell fates (Fig. 5a). Thus, 32Dcl3 cells could be a good model for primary cells in terms of the plasticity of cell fate decision. A cellular library expressing the tyrosine motif-randomized receptors was generated through the same procedure as in the case of Ba/F3 cells, resulting in the estimated transduction efficiency of 1.7%, which was again low enough to secure monoclonal transduction of the library. After culturing the cellular library for 20 days in the presence of the ligand AP20187, we successfully obtained 12 cellular clones. A cell proliferation assay confirmed AP20187-dependent cell proliferation with diverse proliferation levels (Fig. 5b). We chose three fast-proliferating clones (#1, #10, and #12), which had different sequences at the randomized tyrosine motif site as revealed by genomic PCR and subsequent sequencing (Fig. 5c). The three receptor clones were genetically reintroduced into 32Dcl3 cells and compared the cell proliferation levels with the receptor having the signaling domain of the native receptor. Consequently, two out of the three clones (#10 and #12) induced similar levels of AP20187-dependent cell proliferation compared to the native receptor (Fig. 5d). The cell proliferation levels were much higher than those induced by G-CSF, which implies that the motif sequences were phenotypically selected based on fast cell proliferation. Signaling analysis revealed that #10 and #12, which showed the AP20187-dependent strong proliferative activities comparable to the native receptor, activated STAT5, MEK, and Akt in similar or even stronger levels compared to the native receptor (Fig. 5e). On the other hand, the weakly proliferative clone #1 activated only STAT5. The three clones activated STAT3 only weakly but activated STAT5 sufficiently, suggesting that activation of STAT5 may be important for proliferation of 32Dcl3 cells. Stimulation with IL-3, which induces proliferation, strongly activated all signaling molecules tested, whereas stimulation with G-CSF, which induces granulocytic differentiation, activated only STAT3 in sufficient levels. Finally, we examined the expression levels of myeloperoxidase (MPO), which is known as a granulocytic differentiation marker. Consequently, AP20187- and IL-3-stimulated cells did not induce expression of MPO in all clones, while G-CSF-stimulated positive control cells clearly induced expression of MPO (Fig. 5f). These results demonstrate that the cell fate of 32Dcl3 is controlled by the balance of activation of signaling molecules, and that motif sequences that induce a cell fate of interest (*i.e.* proliferation) can be selected from the tyrosine motif library.

## Discussion

In this study, we constructed a tyrosine motif library with randomized sequences at the contact site toward signaling molecules, and demonstrated phenotypic screening of tyrosine motifs that induce cell proliferation. To achieve this, we firstly engineered a receptor scaffold that can activate target signaling molecules in a ligand-dependent manner. Next, we constructed a tyrosine motif library using a negative-control motif as a template, introduced into Ba/F3 cells, and selected motifs that induce cell proliferation directly. Of note, the selected motifs induced similar levels of cell proliferation compared to the cytoplasmic signaling domain of a native receptor. Finally, we applied the motif-screening system to 32Dcl3 cells, which may differentiate or proliferate depending on cytokine signals, and successfully selected motifs that induce proliferation as well. Previous studies on screening tyrosine motifs have focused on in vitro selection of motifs that specifically recruit target signaling molecules^29,30^. To our best knowledge, this is the first report demonstrating phenotypic screening of tyrosine motifs in living cells.

The signaling analyses of the selected tyrosine motif clones demonstrated that proliferation-inducing signaling molecules such as STAT5, MEK, and Akt were activated in both Ba/F3 and 32Dcl3 cells. As shown in our previous study, proliferation of Ba/F3 cells is promoted by STAT3 or STAT5^25,26^. The introductory experiments (Fig. 2) used STAT3 only as a representative of the proliferation-inducing STATs in Ba/F3 cells to validate the receptor scaffold designed. Therefore, it is reasonable that the STAT5-activating chimeric receptors were selected by screening in Ba/F3 cells. Notably, the selected tyrosine motif clones did not activate STAT3 in 32Dcl3 cells, while G-CSF-mediated STAT3 activation triggers granulocytic differentiation in 32Dcl3 cells^31^. Therefore, proliferation-inducing tyrosine motifs were successfully selected even in the cells that may differentiate depending on the signaling properties. While we randomized only the three amino acid residues downstream of the tyrosine residue, the tyrosine motif library containing proliferation-inducing motifs was successfully constructed at least for Ba/F3 and 32Dcl3 cells.

From a viewpoint of the molecular evolution of motifs, one may investigate how the activation patterns of signaling molecules will evolve through further detailed analysis. Phosphoproteomics would be a powerful method to decipher such signaling profiles in a high-throughput fashion. Through detailed analysis of the signaling properties of various motif sequences, one may elucidate not only the activation pattern of signaling molecules that efficiently induce cell fate, but also the mechanism of interactions between tyrosine motifs and their binding domains.

The motif library screening system presented herein would be a new tool for creating designer receptors that efficiently induce cell proliferation. While we applied the system to hematopoietic cells as a proof of concept, the system may also be applied to expansion of various cell types including pluripotent and somatic stem cells. Furthermore, the development of a screening system based on differentiation markers could realize application to not only proliferation but also differentiation. Thus, our approach would open up new possibilities in the field of artificial control of cell fate based on signal transduction engineering.

In this study, we developed a method to screen for synthetic receptors with modified signaling properties that achieve a desired cell fate. In synthetic biology, gene switches that control expression of target genes have mainly been developed to create functional cells. In addition to gene switches, synthetic receptors that endow cells with desired responsiveness to arbitrary external stimuli would become an important tool in synthetic biology. Fusion of synthetic biology tools such as gene switches and synthetic receptors would realize more strictly programmable cell fate control, which would enhance performance of cell-based protein production and cell therapy.

## Materials and methods

### Construction of plasmids

The whole amino acid sequence of each constructed receptor is shown in Supplementary Information (Supplementary Fig. 1). A backbone plasmid pMK-stuffer-IPTG^25^ was used for retroviral infection, in which internal ribosomal entry site (IRES; I) is used for co-expression of the genes encoding a puromycin (P) resistance, a self-cleaving T2A peptide (T), and an enhanced green fluorescence protein (EGFP; G). The open reading frame encoding each receptor sequence was amplified with PCR and inserted into pMK-stuffer-IPTG using In-fusion HD Cloning Kit (TakaraBio). Subcloning, amplification, and sequencing of each plasmid were conducted in a standard protocol.

For construction of a motif library, we chose digestion/ligation procedures as a reliable method. NNS codons were assigned in a PCR primer to randomize 3 amino acid residues downstream of the tyrosine of the STAT1-binding motif. The randomized DNA fragments were generated by PCR using pMK-F1-IPTG, which encodes the engineered receptor with the STAT1-binding motif, as a template, cleaved with *Eco*RI and *Cla*I, and ligated to pMK-F1-IPTG digested with the same enzymes to yield pMK-F1lib-IPTG. MegaX DH10B T1R Electrocomp Cells (ThermoFisher Scientific) were electroporated with pMK-F1lib-IPTG (100 ng) using Gene PulserII (Bio-Rad) set at 2.0 kV, 200 Ω, and 25 µF. All *E. coli* cells were plated on a large-size LB plate to ensure obtaining a sufficient number of antibiotic-resistant colonies exceeding the theoretical DNA library size (3.3 × 10^4^) of the 3 amino-acid randomized mutations created by degenerate NNS codons. The colonies were harvested to extract plasmids using QIAGEN Plasmid Midi Kit (Qiagen).

### Clonal vector transduction

For transduction of Ba/F3 cells (RIKEN Cell Bank #RCB0805, Ibaraki, Japan), retroviral packaging Plat-E cells (kindly provided by Dr. T. Kitamura, The University of Tokyo) were seeded on 6-well plates a day before transfection. On the next day, 3 µg of a transgene plasmid, 3 µL of PLUS Reagent (ThermoFisher Scientific), and 150 µL of Opti-MEM (ThermoFisher Scientific) were mixed in a tube, while in another tube 7.5 µL of Lipofectamine LTX Reagent (ThermoFisher Scientific) and 150 µL of Opti-MEM were mixed. Then, the mixtures derived from the two tubes were mixed together and added to the pre-cultured Plat-E cells (day 0). The culture medium was changed on day 1. On day 2, the retroviral supernatant was harvested, filtered with a 0.45 µm PVDF membrane, added to Retronectin (TakaraBio)-coated 24-well plates, and left for 6 h at room temperature. Retronectin can co-localize cells and retroviral particles, leading to high transduction efficiency and thus shortening the time required for obtaining a sufficient cell number of transductants for further analyses. After washing with PBS once, Ba/F3 cells (1.0 × 10^5^ cells/well) were seeded on the plates, followed by addition of 1 µg/mL of puromycin on day 3 or 4 to select stably transduced cells.

Ba/F3 cells can be transduced with retroviral particles with the ecotropic envelope, whereas 32Dcl3 cells (RIKEN Cell Bank #RCB1377) cannot. Therefore, 32Dcl3 cells were transduced with retroviral particles pseudotyped with the VSV-G envelope. For transduction of 32Dcl3 cells, 293GP cells (kindly provided by Dr. M. Otsu, Kitasato University) were used for pseudotype retroviral packaging^9^. The transfection protocol was the same as in the case of Ba/F3 cells, except 2.25 µg of a transgene plasmid and 0.75 µg of pCMV-VSV-G were used. On day 2, the retroviral supernatant was centrifuged at 6000 g for 16 h at 4 °C and concentrated 100-fold with RPMI medium as a solvent. The concentrated virus was added to 32Dcl3 cells (1.0 × 10^5^ cells) with 10 µg/mL of protamine sulfate, followed by addition of 3 µg/mL of puromycin on day 3 or 4 to select stably transduced cells.

### Library vector transduction

In the library vector transduction, the following retroviral transduction protocol using a cationic reagent protamine sulfate empirically results in transduction efficiency of < 10%, which is desirable for monoclonal library transduction into recipient cells. Overall procedures were approximately the same as in the case of clonal vector transduction, except the cell cultures and reagents used were scaled up. The scaled-up values in library vector transduction are listed as follows.

For transduction of Ba/F3 cells, Plat-E cells were seeded on a 10 cm dish; plasmid solution was 16 µg of a transgene plasmid, 32 µL of P3000 Reagent (ThermoFisher Scientific), and 500 µL of Opti-MEM; lipofectamine solution was 21.7 µL of Lipofectamine 3000 Reagent (ThermoFisher Scientific) and 500 µL of Opti-MEM; 1.0 × 10^7^ of Ba/F3 cells were transduced in the presence of 10 µg/mL of protamine sulfate.

For transduction of 32Dcl3 cells, 293GP cells were seeded on a 10 cm dish; plasmid solution was 10 µg of a transgene plasmid, 2.5 µg of pCMV-VSV-G, 12.5 µg of PLUS Reagent, and 1 mL of Opti-MEM; lipofectamine solution was 31 µL of Lipofectamine LTX Reagent and 2.4 mL of Opti-MEM; 1.0 × 10^7^ of 32Dcl3 cells were transduced in the presence of 10 µg/mL of protamine sulfate.

### Flow cytometry

EGFP positive rates were measured with FACSCalibur (BD Biosciences).

### Library screening

For screening of Ba/F3 transductants, transduced cells (1.1 × 10^5^) were seeded on 96-well plates at 1.0 × 10^3^ cells/mL with 10 nM AP20187 (TakaraBio) on day 0. To aid clonal cell growth in the limiting-diluted condition, non-transduced parental cells (3.0 × 10^5^ cells/mL) were mixed as feeder cells. The growing colonies were scaled up into 24-well plates and subsequently 6 cm dishes. Finally, the fastest 18 cellular clones were subjected to further assays. Genomic DNA was extracted with DNeasy Blood & Tissue Kit (QIAGEN). Genomic PCR was conducted to amplify the randomized region. The amplified fragments were subjected to sequencing. The same procedures were conducted for screening of 32Dcl3 transductants.

### Cell proliferation assay

Cells were washed with PBS twice and seeded into 24-well plates in the culture medium containing various concentrations of AP20187 (TakaraBio), 1 ng/mL of IL-3 (ThermoFisher Scientific), or 10 ng/mL of G-CSF (R&D Systems). Viable cell densities were estimated by Cell Counting Kit-8 (Dojindo Laboratories) by measuring absorbance at 450 nm using GloMax Discover Microplate Reader (Promega).

### Western blotting

The detailed experimental procedures for western blotting were described previously^32^. In signaling analyses, cells were cultured in the medium devoid of IL-3 for 10–16 h, and subsequently stimulated with 10 nM AP20187, 1 ng/ml IL-3, or 10 ng/ml G-CSF at 37˚C for 15 min. In the MPO expression assay, cells were cultured in the medium containing 10 nM AP20187, 1 ng/ml IL-3, or 10 ng/ml G-CSF for 5 days. Proteins of the cell lysates were resolved by SDS-PAGE, transferred on nitrocellulose membranes, and probed with primary and secondary antibodies. The antibodies used are listed in Supplementary Information (Supplementary Fig. 2).

### Supplementary Information

Supplementary Information.

Supplementary Figures.

## Data Availability

All data generated or analyzed during this study are included in this published article and its Supplementary Information.
