# Supplementary material for: Phenotypic screening of signaling motifs that efficiently induce cell proliferation
Source: Sci Rep. 2023 Sep 20;13:15639. doi: 10.1038/s41598-023-42378-6 (PMC10511696; doi:10.1038/s41598-023-42378-6)
Supplement: Supplementary file 2 — Supplementary Figures. [file 41598_2023_42378_MOESM2_ESM.pdf]

Supplementary Information

Phenotypic screening of signaling motifs that efficiently induce cell proliferation

Kirato Umene[1], Teruyuki Nagamune[1], Masahiro Kawahara[1,2,*]

[1]Department of Chemistry and Biotechnology, Graduate School of Engineering, The University of Tokyo, 7-3-1 Hongo, Bunkyo-ku, Tokyo 113-8656, Japan.

[2]Laboratory of Cell Vaccine, Microbial Research Center for Health and Medicine (MRCHM), National Institutes of Biomedical Innovation, Health and Nutrition (NIBIOHN), 7-6-8 Saito-Asagi, Ibaraki-shi, Osaka 567-0085, Japan.

[*]Correspondence: Masahiro Kawahara, Laboratory of Cell Vaccine, Microbial Research Center for Health and Medicine (MRCHM), National Institutes of Biomedical Innovation, Health and Nutrition (NIBIOHN), 7-6-8 Saito-Asagi, Ibaraki-shi, Osaka 567-0085, Japan.
E-mail: m-kawahara@nibiohn.go.jp

a) Motif chimera

MGSSKSKPKDPSQRGSGGVQVETISPGDGRTFPKRGQTCVVHYTGMLEDGKKVDSS
RDRNKPFKFMLGKQEVIRGWEEGVAQMSVGQRAKLTISPDYAYGATGHPGIIPPHATL
VFDVELLKLEGSGRWQFPAHYRRLRHALWPSLPDLHRVLGQYLRDTAALSPPKATVS
DTCEEVEPSLLEILPKSSERTPLPLRVGGGGSGGGGSGGGGSRVmotif_sequenceIDEQK
LISEEDL

STAT1-binding motif
PTSFGYDKPHVL

STAT3-binding motif
VVHSGYRHQVPS

b) Mpl chimera

MGSSKSKPKDPSQRGSGGVQVETISPGDGRTFPKRGQTCVVHYTGMLEDGKKVDSS
RDRNKPFKFMLGKQEVIRGWEEGVAQMSVGQRAKLTISPDYAYGATGHPGIIPPHATL
VFDVELLKLEGSGRWQFPAHYRRLRHALWPSLPDLHRVLGQYLRDTAALSPPKATVS
DTCEEVEPSLLEILPKSSERTPLPLCSSQAQMDYRRLQPSCLGTMPLSVCPPMAESGSC
CTTHIANHSYLPLSYWQQPIDEQKLISEEDL

Supplementary Figure 1 The amino acid sequences of the motif and mpl chimeras. Purple: myristoylation signal. Black: linker or extra sequence. Green: $FKBP_{F36V}$. Blue: truncated (the JAK-binding domain) or whole intracellular domain of c-mpl. Brown: Myc tag. The full plasmid sequences for the motif chimera (incorporating the STAT1-binding motif) and the mpl chimera are provided as annotated .gbk files as Supplementary Material.

Antibody	Manufacturer	Cat#
rabbit anti-phospho-STAT1(Y701)	Cell Signaling Technology	9167
rabbit anti-STAT1	Cell Signaling Technology	9172
rabbit anti-phospho-STAT3 (Y705)	Cell Signaling Technology	9145
rabbit anti-STAT3	Santa Cruz Biotechnology	sc-482
rabbit anti-phospho-STAT5(Y694)	Cell Signaling Technology	9351
rabbit anti-STAT5	Santa Cruz Biotechnology	sc-835
rabbit anti-phospho-Akt (T308)	Cell Signaling Technology	13038
rabbit anti-Akt	Cell Signaling Technology	9272
rabbit anti-phospho-MEK1/2 (S217/221)	Cell Signaling Technology	9154
rabbit anti-MEK1/2	Cell Signaling Technology	8727
rabbit anti-c-myc tag	Bethyl Laboratories	A190-105A
rabbit anti-GAPDH	Cell Signaling Technology	5174
rabbit anti-MPO	Santa Cruz Biotechnology	sc-16128-R
HRP-conjugated goat anti-rabbit IgG	Thermo Fisher Scientific	G-21234

Supplementary Figure 2 The antibodies used in Western blotting.

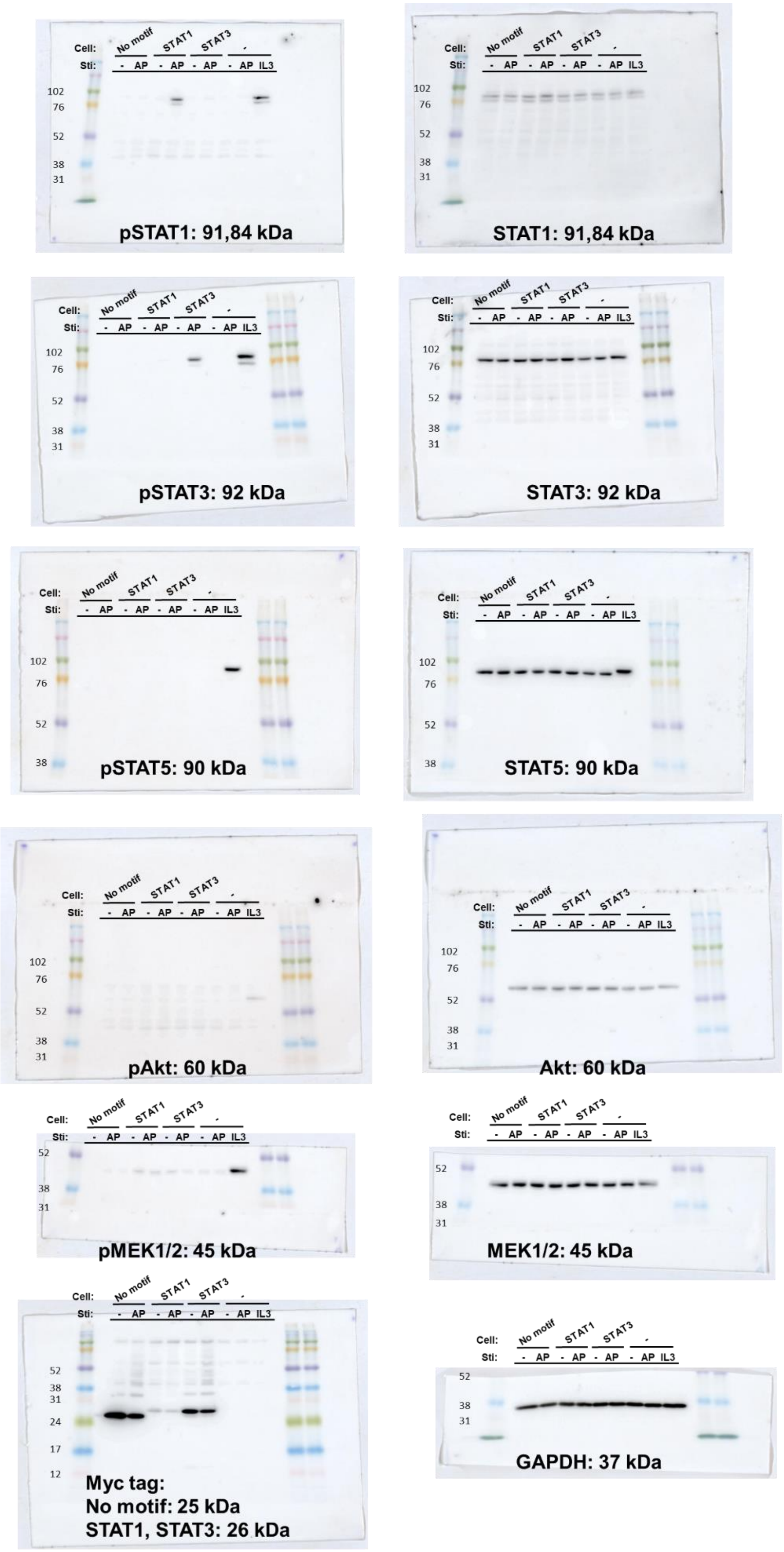

Supplementary Figure 3. Uncropped blot images for Fig. 2b.

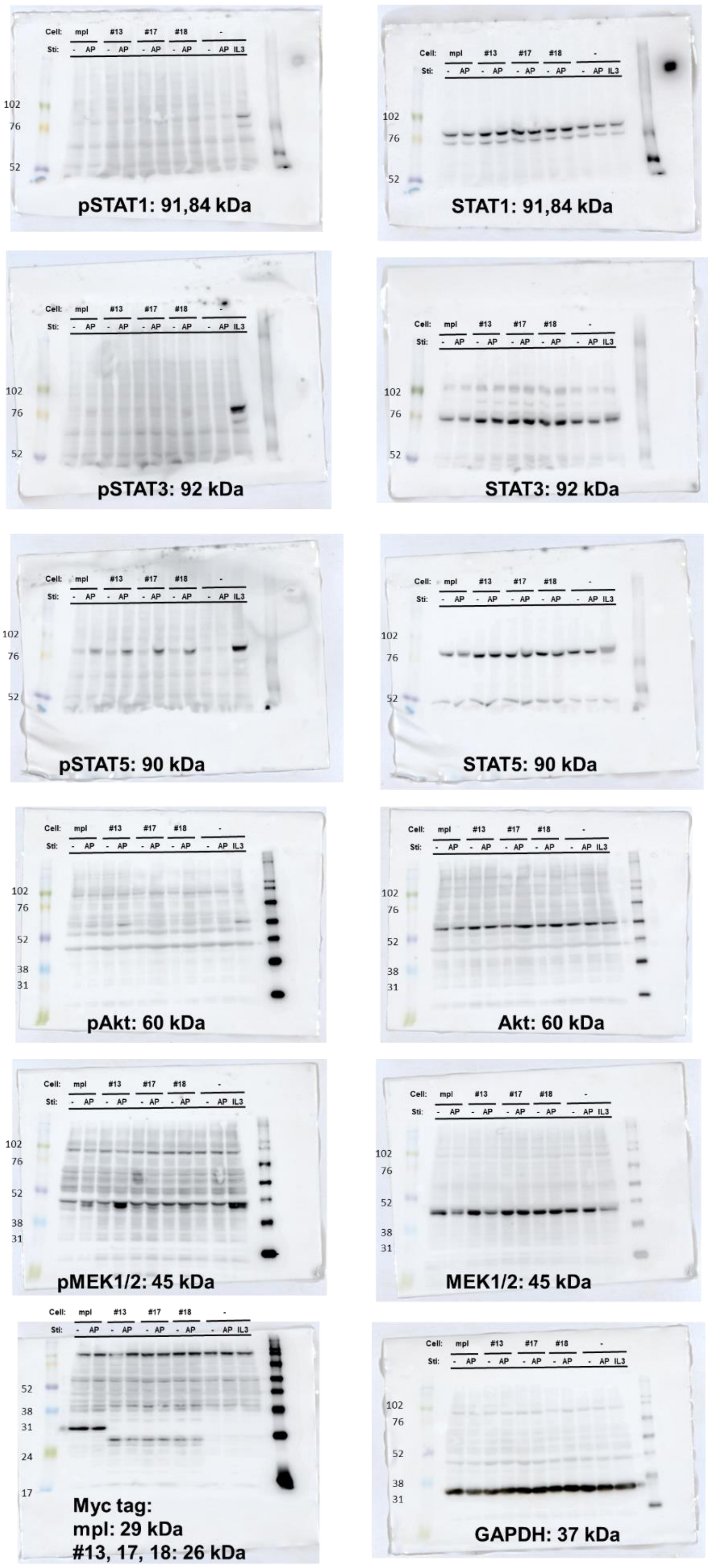

Supplementary Figure 4. Uncropped blot images for Fig. 4d.

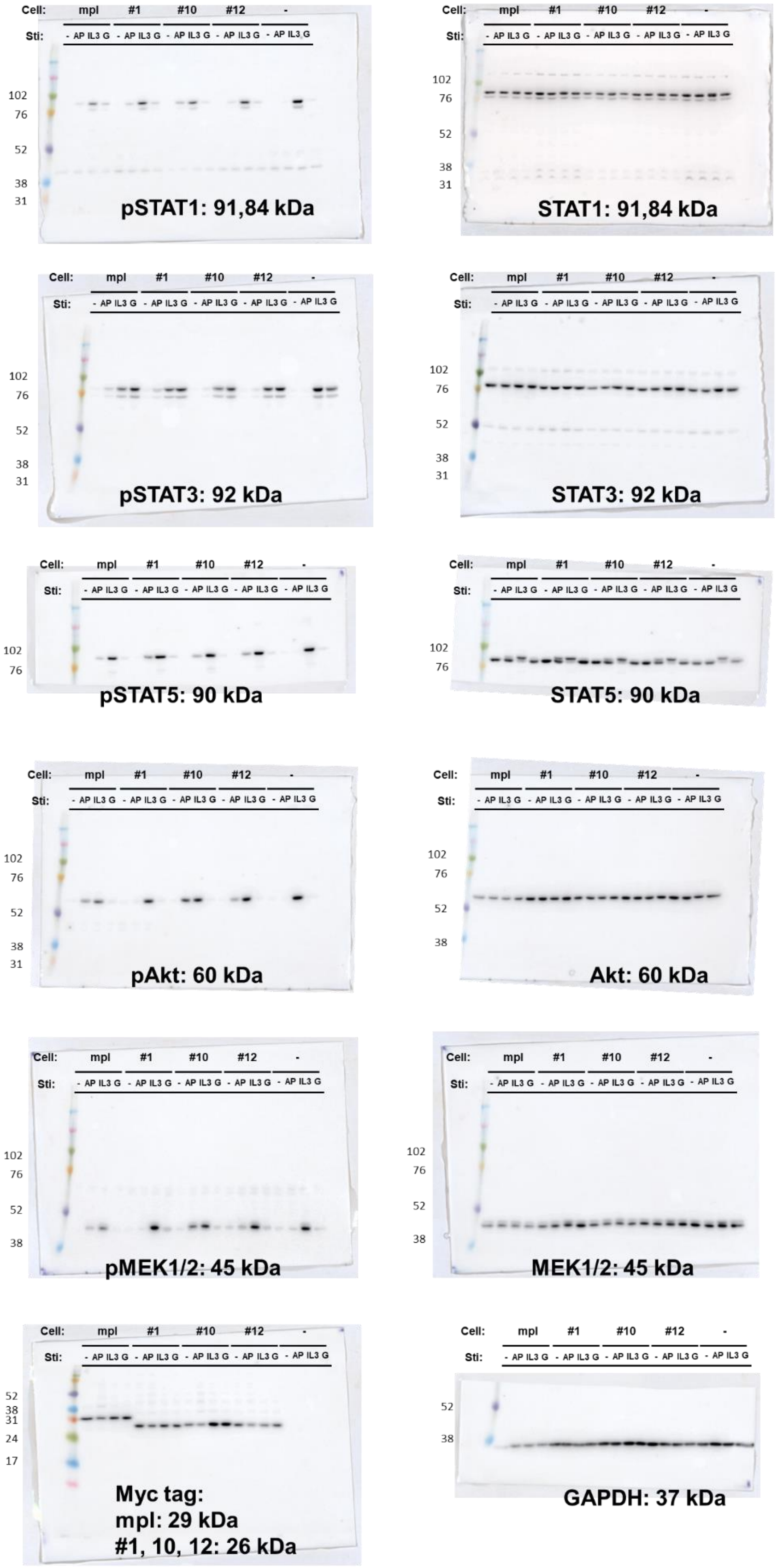

Supplementary Figure 5. Uncropped blot images for Fig. 5e.

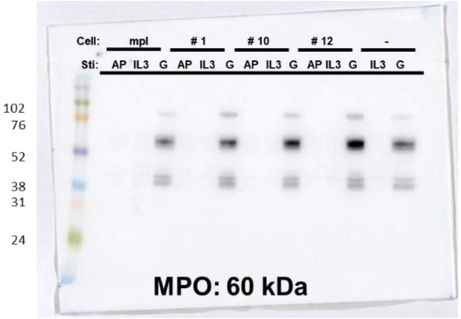

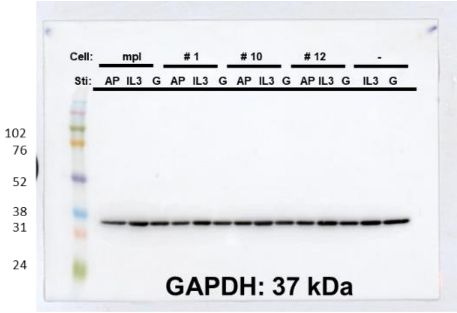

Supplementary Figure 6. Uncropped blot images for Fig. 5f.
